# Germany and the United States in coronavirus distress: internal versus external labour market flexibility

**DOI:** 10.1186/s12651-022-00316-5

**Published:** 2022-08-10

**Authors:** Alexander Herzog-Stein, Patrick Nüß, Lennert Peede, Ulrike Stein

**Affiliations:** 1 Macroeconomic Policy Institute (IMK), Georg-Glock-Str. 18, 40474 Düsseldorf, Germany; 2 University of Koblenz-Landau, 76829 Landau, Germany; 3 Kiel University, Wilhelm-Seelig-Platz 1, 24118 Kiel, Germany; 4 University of Muenster, Schlossplatz 2, 48149 Münster, Germany

**Keywords:** Working-time reduction, Safeguarding employment, Unemployment, Internal flexibility, External flexibility, Short-time work, Temporary layoffs, Great recession, Coronavirus recession, Covid-19 pandemic, E24, E32, J08, J20

## Abstract

Germany and the United States pursued different economic strategies to minimise the impact of the Coronavirus Crisis on the labour market. Germany focused on safeguarding existing jobs through the use of internal flexibility measures, especially short-time work (STW). The United States relied on a mix of external flexibility and income protection. On this basis, we use macroeconomic time series to examine the German strategy of securing employment through internal flexibility by contrasting it with the chosen strategy in the United States. In Germany, temporary cyclical reductions in working hours are mainly driven via STW. US unemployment rose at an unprecedented rate, but unlike in previous recessions, it was mostly driven by temporary layoffs. However, a closer look at the blind spots of the chosen strategies in both countries showed that despite the different approaches, people in weaker labour market positions were less well protected by the chosen strategies.

## Introduction

During both the Great Recession and the Coronavirus Recession, unemployment in Germany increased only moderately, especially given the severity of these recessions. However, in both recessions there were instead significant temporary reductions in working time by means of internal flexibility, i.e., the internal adjustment of the labour input used in the production process along the intensive margin especially through the use of short-time work (STW). While most European countries have also relied on STW to tackle the crisis, in the United States external flexibility was dominant, i.e., the adjustment of labour input via the external labour market, and there was a sharp temporary increase in unemployment of historic proportions.^1^ Instead of labour hoarding through STW programmes, the United States decided to insure worker’s incomes with instruments such as cash transfers and temporary increases in unemployment benefits instead of protecting employment.

Giupponi et al. (2022) discuss extensively upsides and downsides of both strategies. We contribute to this debate by providing a detailed descriptive analysis of the German strategy of safeguarding employment via internal flexibility by contrasting it with the strategy chosen in the United States and the labour market experience in the two countries in 2020.

For Germany, we show that despite the dramatic decrease in real gross domestic product (GDP), unemployment only increased moderately. While during the Great Recession all working-time instruments contributed to the reduction in working time, STW now accounts for almost all of the working-time reduction as the government focused on this instrument to maintain employment. In contrast, the United States experienced a comparable decrease in real GDP, but also a temporary increase in unemployment on an unprecedented scale. However, the nature of unemployment changed as well. In contrast to the Great Recession, temporary layoffs during the Coronavirus Recession played a dominant role in the United States and allowed for a fast recovery of the unemployment rate. While both countries approached the crisis differently, remarkably their weakness is the same. The methods to secure employment and income were less pronounced for individuals in weaker positions on the labour market (atypical employment and low wage earners).

The remainder is structured as follows: In Section 2 we provide a concise summary of how the German labour market was affected by the Coronavirus Crisis relative to the experience in the United States and describe the differing labour-market related policy responses in the two countries. Section 3 presents a comparative business-cycle analysis of the Coronavirus Recession and the Great Recession in Germany as well as in the United States and has a closer look at the country specific margins of labour market adjustments. Then, in Section 4 we investigate potential blind spots of the chosen policies in the two countries. Section 4.1 concludes.

## The German and US labour market during the Covid-19 pandemic

**Fig. 1:**
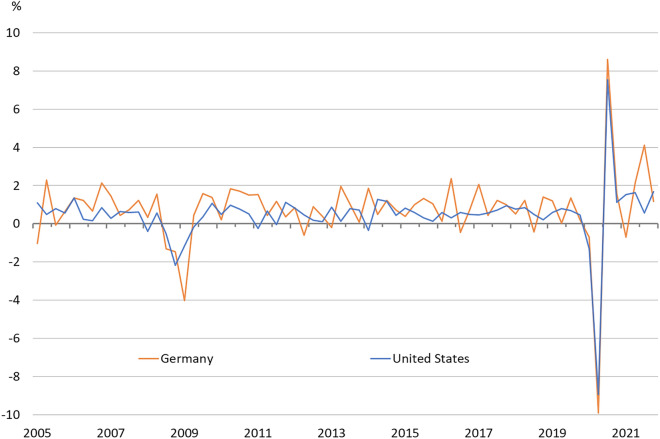
Real gross domestic product in Germany and the United States (2005–2021). Quarterly change in real GDP; seasonally adjusted. Sources: Federal Statistical Office (Destatis); Bureau of Economic Analysis; authors' calculations

The outbreak of the global Covid-19 pandemic and its economic impact on the world economy caused a major economic crisis. Both the German and the US economy were severely affected by the pandemic and experienced economic contractions of similar magnitude. The German economy started to be severely affected by the pandemic at the end of the first quarter 2020 and went into a partial lockdown from mid-March to May 2020. The result was an economic slump of historic proportions. In the second quarter 2020, real GDP contracted by 9.9%, after it fell already by 0.7% in the first quarter 2020 (Fig. 1). In total in the first two quarters 2020 real GDP fell by 10.5% seasonally adjusted. The United States declared a public health emergency at the End of January 2020 and in mid-March 2020 a national emergency in response to the Covid-19 pandemic. From Spring 2020 onwards there were also widespread business closures and social distancing practices (Houseman 2022). Real GDP fell seasonally adjusted by 8.9% in the second quarter 2020 after a 1.3% decrease in the first quarter.

**Fig. 2:**
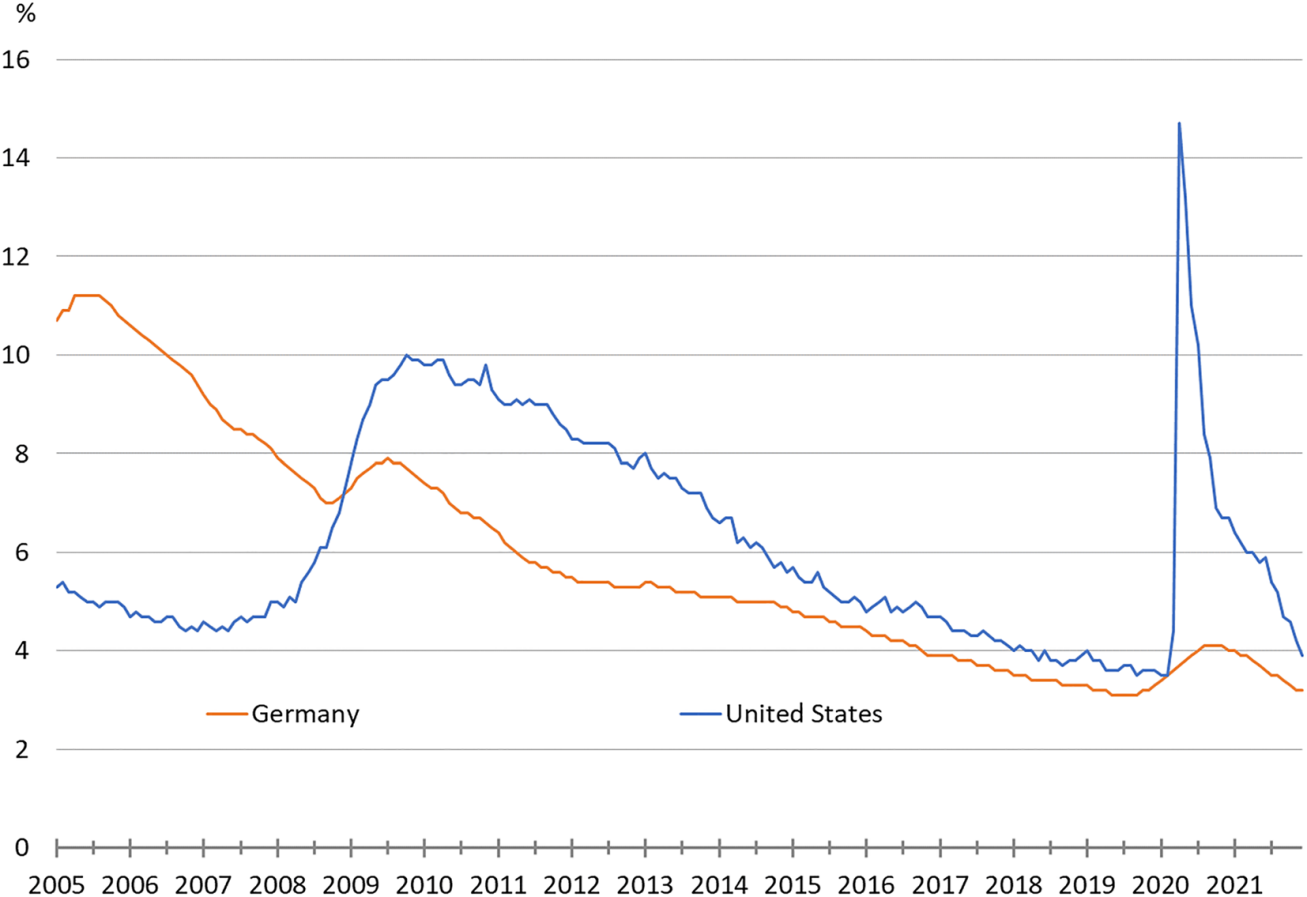
Unemployment rates in Germany and the United States (2005 to 2021). The definition used in the Labour Force Survey follows the definition of the Internal Labour Organization. Number of all unemployed people aged 15–74 as percentage of the labour force. Sources: Eurostat; own presentation

The Coronavirus Crisis had a marked impact on labour market performance in Germany and the United States. Unemployment increased markedly in both countries (Fig. 2).^2^ However, the magnitude of the rise in unemployment in the two countries was different.

While in the United States the unemployment rate increased dramatically from 3.5 to 14.7% from February to April 2020, in Germany the rise in unemployment was much less pronounced but went on for longer. From February to August 2020 the German unemployment rate increased from 3.5 to 4.1% and started only to decline again in December 2020. In contrast, in the Unites States unemployment declined relatively fast and continuously after its peak in April 2020. In addition, the US civilian labour force declined by 5.0% from February to April 2020, while in Germany the labour force decrease from February to May 2020 was much smaller at 1.5%.

In comparison to the Great Recession, the increase in the US unemployment rate from its minimum was around twice as large in the Coronavirus Recession, but the following decline was much quicker this time. In Germany, unemployment rose faster this time than in the Great Recession, when unemployment started to rise in November 2008 and peaked in July 2009 (see Fig. 2). With a total increase of 0.6 percentage points the rise in unemployment was now slightly less than at that time (+ 0.9 percentage points). Compared to the United States and against the backdrop of the massive decline in economic activity the rise in unemployment was in both downturns relatively moderate in Germany.

The differences in the development of unemployment in the two countries in the Coronavirus Recession are quite remarkable. But the policy responses in Germany and the United States regarding the labour market as a result of the economic impact of the pandemic were also quite different. Table 1 contrasts the discretionary policy changes in Germany with those in the United States in 2020 sorted into four categories: legislative changes regarding the respective STW scheme, the provision of business support, the expansion of unemployment benefits, and the provision of income support for households. STW and business support reflect measures that incentivise the use of internal flexibility to safeguarding employment. Expansions of unemployment benefits and income support for households aim at insuring incomes given that establishments adjust labour input along the extensive margin.

**Table 1:** Discretionary policy changes during the Coronavirus Crisis in the United States and Germany (dating corresponds to date of passage)

Germany	United States
**2020Q1**	
Short-time work • Simplified eligibility criteria • 100% reimbursement of social insurance contributions for hours affected by STW • Expansion of additional income opportunities during STW	Short-time work • Federal financial support for states’ STC schemes
Business support • *Soforthilfen: g*rants for small businesses & self-employed, administrated by *Länder* • Loans and credit guarantees	Business support • *Paycheck Protection Program (PPP)*: forgivable loans to SME • *Employee Retention Tax Credit*: payroll tax credit for employers • Tax credits for obliged paid leave by employees • *Disaster Loans Program*: Federal Funding of loans from Small Businesses Administration
Unemployment benefits • Simplified eligibility criteria for basic income support (ALG II) (e.g., further inclusion of self-employed, suspension of means testing)	Unemployment benefits • *Federal Pandemic Unemployment Compensation* (*FPUC*): additional benefit of $600 per week until end of July • *Pandemic Emergency Unemployment Compensation* (*PEUC*): extension of max. eligibility period • *Pandemic Unemployment Assistance* (*PUA*): simplified eligibility criteria (e.g., inclusion of self-employed)
Income support (households) • Simplified eligibility criteria for child supplements • Compensation for earnings losses due to child care	Income support (households) • Child Tax Credits • Economic Impact Payments: Stimulus checks for households per eligible adult ($1200) and child ($500) • Obligation to SME to provide paid sick leave, paid family leave, paid medical leave
**﻿2020Q2**	
Short-time work • Extension of maximum eligibility period • Temporary increase in replacement rates until end of 2020	Business support • Improved conditions regarding loans and forgiveness of loans (June) • Amendment of PPP: Provision of additional $320 bn. (April)
Unemployment benefits • Expansion of max. eligibility period for unemployment insurance (ALG I) by three months	
**2020Q3**	
Business support • Grants for SME & self-employed *(Überbrückungshilfe)* • Hiring credit for apprentices for SME of €2000	Unemployment benefits • Extension and modification of expiring *FPUC*: $300 per week and conditioning eligibility on receiving at least $100 from other state unemployment benefits
Unemployment benefits •Extension of simplified eligibility criteria for ALG II	Income support (households) •Provision of assistance to renters and homeowners
Income support (households) • Stimulus Checks for families: €300 per child (*Kinderbonus*) • Tax Credits for single parents • Expansion of max. eligibility period for elderly/child care compensation • Temporary VAT reduction	
**2020Q4**	
Short-time work • Extension of the key measures until end of 2021	Business support • Modification & extension of different programs (i.a., *the Employee Retention Tax Credit*, *PPP*, etc.)
Business support • Grants for foregone revenues *(November-/Dezemberhilfen)* • Grants for SME *(Überbrückungshilfe II)*	Unemployment benefits • *FPUC*: $300 per week until March 2021 • *PEUC* applies until March 2021, expansion of max. eligibility period to 24 weeks • *PUA* applies until March 2021; expansion of max. eligibility period to 50 weeks
	Income support (households) • Economic Impact Payments for adults ($600) and children ($600)

Sources: Federal Ministry of Labour and Social Affairs, Federal Ministry of Finance, Steffen (2021) for Germany; U.S. Government, Chetty et al. (2020), Houseman (2022) for United States

In short, while Germany put a focus on subsidising the use of internal flexibility, especially by providing more generous STW allowance, the United States put a strong focus on insuring incomes by providing generous unemployment benefits and allow for adjustments along the extensive margin.^3^

Policy measures to expand the use of STW had only a relevant impact in Germany. In line with the empirical evidence on the effectiveness of the rule-based and the discretionary component of STW in safeguarding employment in the Great Recession (Balleer et al. 2019; Gehrke and Hochmuth 2021), discretionary policy changes made the use of STW in the pandemic more attractive. The similarity of the changes in both crises is striking (Herzog-Stein et al. 2021), albeit the extensions were much faster and more generous this time. Crucial for the increased take up of STW were the extension of the eligibility period of STW and the simplified eligibility criteria with respect to the scope of STW in March 2020. Moreover, immediately a full reimbursement of social security contributions for hours affected by STW was introduced to reduce residual costs of companies when using STW. Thus, strong incentives for companies to use STW were created.

In contrast, in the United States, STW measures mainly consist of financial support for state-level STW schemes by the federal level. Moreover, only 26 states had STW schemes at the beginning of the crisis implemented (Houseman 2022).

Instead of subsidizing working-time reductions by expanding the STW schemes, the US government aimed at subsidised labour hoarding by providing business support especially with the CARES Act in March 2020. These measures aimed at incentivising businesses to retain their employees, too. Major elements of the CARES Act were the Paycheck Protection Program (PPP), which mainly entailed forgivable loans to SME, and the Employee Retention Tax Credits. However, as Autor et al. (2022) show, 66 to 77% of the issued loans in 2020 do not seem to actually have been used to retain their employees but were kept by business owners and shareholders. Both measures, the PPP and the Employee Retention Tax Credits were amended in subsequent laws later in 2020. Overall, these two measures of business support from the CARES Act account for $861 billion or 4.1% of nominal GDP in 2020 (CRFB 2021).

In Germany all programs providing business support in 2020 account for €54.73 billion or 1.6% of nominal GDP in 2020 (Federal Ministry of Finance 2021). The main programs at the federal level are the *Soforthilfen* in March, the *Überbrückungshilfen I–III* from the Stabilizing Package in July, and the *November-/Dezemberhilfen*. These programs contained grants and forgivable loans.

Apart from the differences in incentivising the use of measures of internal flexibility between Germany and the United States there are also differences in the extent of insuring workers’ incomes in case establishments adjust their labour input along the extensive margin. The CARES Act introduced additional federal unemployment benefits (FPUC) of $600 per week until July 2020. In further programs the maximum eligibility period and eligibility of further worker groups has been introduced. In August the FPUC was replaced by $300 per week, and in the Consolidated Appropriations Act the maximum eligibility period was further expanded.

In contrast to the US, the discretionary changes in unemployment benefits in Germany had only minor relevance in insuring incomes of the unemployed. In the first *Social Security Package* in March 2020 eligibility criteria for basic income support (ALG II) were simplified. In the subsequent second *Social Security Package* from May 2020 the maximum eligibility period for unemployment benefits was expanded by three months.

Besides insuring incomes with unemployment benefits, stimulus payments are of particular importance to stabilize incomes. In Germany, the Stabilizing package contains a stimulus check for families by providing €300 per child and tax credits for single parents. Still, they are by far not as expansive as the Economic Impact Payments in the US. The CARES Act entailed so-called Economic Impact Payments of up to $1200 per adult for eligible individuals (earning less than $75,000) and $500 per qualifying child under age 17. Additionally, a child tax credit was implemented.

## Germany and the United States: internal versus external flexibility in the Covid-19 pandemic

To gain a better understanding of the impact of the different policy strategies in Germany and the United States on the labour market, we conduct a business-cycle analysis comparing, on the one hand, the Coronavirus Recession with the Great Recession in both countries and contrasting the German experience with that of the United States on the other hand. Then, we look at the case of Germany in the Coronavirus Recession with particular interest in the relative importance of working-time instruments (overtime, regular working time, working time accounts (WTA), and STW) in safeguarding employment, before examining the role of external flexibility in the United States via temporary and permanent lay-offs during the Covid-19 pandemic.

### Germany and the United States: a business cycle analysis^4^

For a business-cycle analysis of the cyclical variations in economic activity, employment, productivity, and working hours in these two countries, we must first determine the peak and trough of the Great Recession and the latest recession in Germany and the United States. We follow the business cycle dating of the NBER’s Business Cycle Dating Committee for the United States and of the German Council of Economic Experts (GCEE) for Germany.

In Germany the economic downturn of the Great Recession started after the first quarter of 2008 (peak) and ended in the second quarter 2009 (trough). According to the NBER, in the United States it started already after the fourth quarter 2007 (peak) and also ended in the second quarter 2009 (Table 2). As for the latest downturn, the Coronavirus Recession, in both countries economic activity peaked in the fourth quarter 2019.^5^ The NBER dated the trough of economic activity to the second quarter 2020. For Germany, the GCEE has not yet determined the trough of economic activity. However, the development of economic activity in Germany in 2020 and 2021, especially GDP growth, also points towards the second quarter 2020 as time of the economic trough. Therefore, in the further analysis we assume that 2020q2 is also the trough of economic activity in Germany, but present data for both countries until the end of 2021.

**Table 2:** Dating the Great Recession and the Coronavirus Recession in Germany and the United States

Contractions	Germany		United States	
	Peak quarter	Trough quarter	Peak quarter	Trough quarter
Great Recession	2008 Q1	2009 Q2	2007 Q4	2009 Q2
Coronavirus Recession	2019 Q4	Not yet determined	2019 Q4	2020 Q2

Sources: German Council of Economic Experts (GCEE); NBER’s Business Cycle Dating Committee

Given the determination of the Great Recession and the Coronavirus Recession in Germany and the United States by the GCEE and the NBER, respectively, we focus on cyclical variations in economic activity in the following business-cycle analysis. Therefore, we extract the cyclical and trend component using the Hodrick-Prescott Filter (HP-Filter). As is common practice for quarterly data, we use a HP-Filter with a smoothing parameter $\lambda$ equal to 1600 to detrend the quarterly time series from 1991 to 2021. It is well known that the HP-Filter, like other filter methods, suffers from an end-point problem. Since our main focus is on the slump until 2020q2 and we use six additional data points, the impact of this end-point problem is still there but of a smaller importance for the analysis of the recession periods. However, we are careful in interpreting results after 2020q2 and closer to the end of the data set. For completeness and clarity, we present results up to 2021q4, the end of our dataset.

**Fig. 3:**
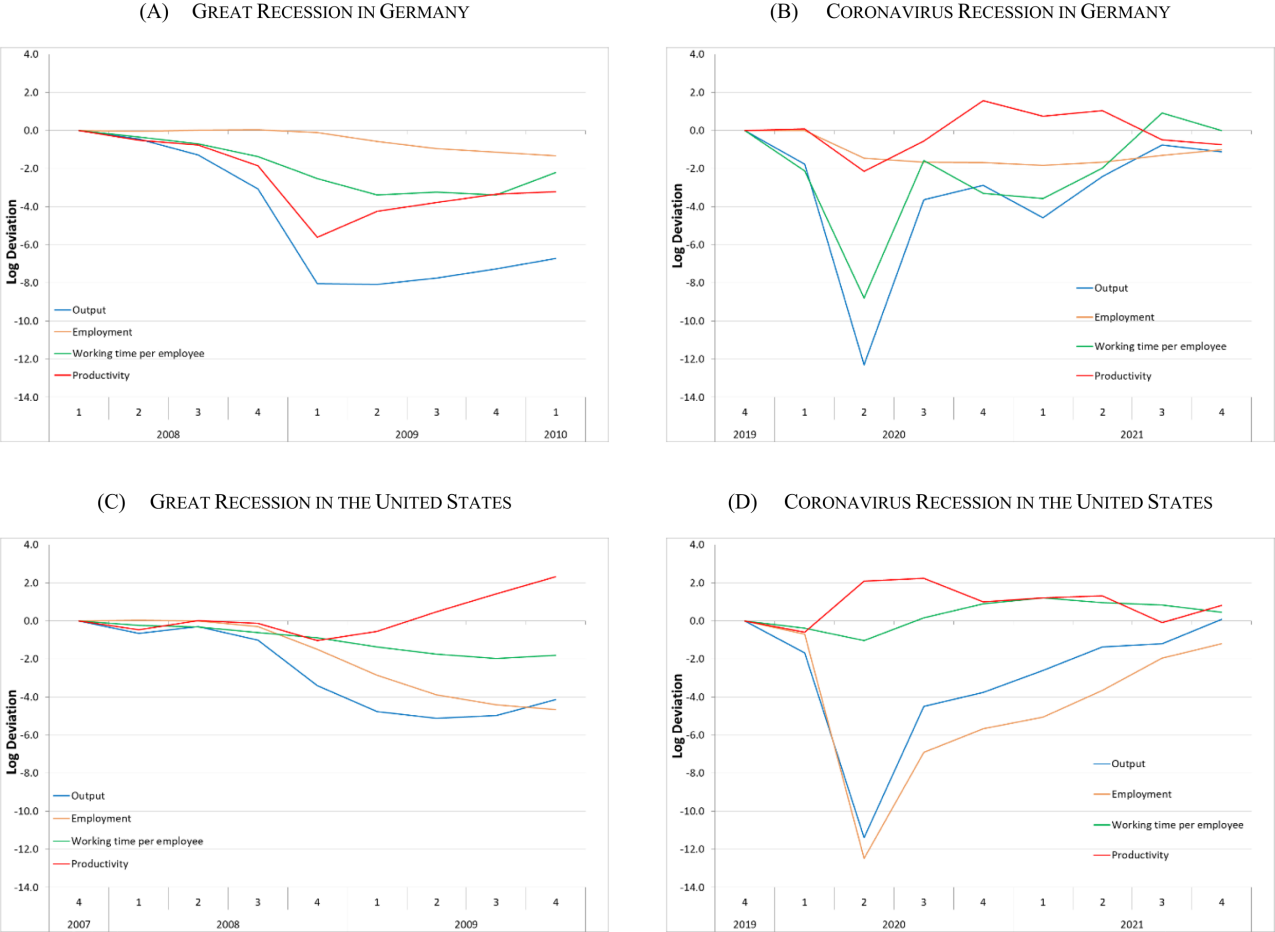
Great Recession vs. Coronavirus Recession in Germany and the United States. Log deviations from peak quarter (Germany: 2008q1 respectively 2019q4; United States: 2007q4 respectively 2019q4) measured in log points. Output (= real GDP), employment, working time in hours worked per quarter per employee, and productivity (= labour productivity per hours worked) are seasonally and/or calendar adjusted. Sources: Federal Statistical Office (Destatis); Bureau of Economic Analysis; U.S. Bureau of Labor Statistics (BLS); own calculations

For Germany and the United States, Fig. 3 examines the economic dynamics of the cyclical components of real GDP, employment, productivity and working time, i.e., average hours worked per employee, during the Great Recession (Germany: Panel A, and United States: Panel C) and the Coronavirus Recession (Germany: Panel B, and United States: Panel D). All figures are normalised to the respective beginning of the two economic downturns, i.e., for the Great Recession 2008q1 for Germany and 2007q4 for the United States, and for the Coronavirus Recession 2019q4 for both economies.

Due to the economic shock caused by the Covid-19 pandemic, the Coronavirus Recession was much more severe. From 2019q4 to 2020q2, cyclical real GDP contracted by 12.3% in Germany and by 11.4% in the United States as a direct consequence of the Covid-19 pandemic. In the Great Recession, the corresponding cyclical decline in output from peak to trough was 8.1 and 5.1%, respectively.

As in the Great Recession, most of the economic shock in Germany was absorbed by internal flexibility in the labour market via a temporary working-time reduction and labour hoarding in the form of a procyclical decline in labour productivity. However, this time the relative contribution of internal flexibility was even larger than in the Great Recession. From peak to trough, the cyclical reduction in the average number of hours worked per employee was twice as high as in the Great Recession (− 8.8 vs. − 3.4%). In Germany, productivity reacted much stronger in the Great Recession than in the Coronavirus Recession (− 5.6 vs. − 2.1%). Even though speed and intensity of job losses were more pronounced in the Coronavirus Recession, in both economic recessions cyclical employment continued to decline, even after the trough of the business cycle. Overall, employment declined cyclically by 0.6% from 2008q1 to 2009q2; by 2010q1 it had fallen by a further 0.7%. Thereafter, cyclical employment started to recover. In the Coronavirus Recession, cyclical employment declined by 1.4% from 2019q4 to 2020q2 and a further 0.4% by 2021q1. It then started to recover over the remaining quarters of 2021.

In contrast to the economic developments observed in Germany, in the United States external flexibility bore the brunt of adjustment in response to the economic shock as a consequence of the Covid-19 pandemic (Panel D). From peak to trough, cyclical employment decreased by 13%. However, in contrast to the cyclical development in Germany, employment then started to recover. This also contrasts the cyclical behaviour of employment in the Great Recession when it continued to decline beyond the trough quarter.

Average hours worked per employee decreased by 1.0% in the Coronavirus Recession, while labour productivity cyclically increased by 2.1% from peak to trough. This also contrasts with developments in the United States during the Great Recession, when internal flexibility from cyclical reductions in working hours per worker and changes in labour productivity together accounted for about a quarter (− 1.2%) of the labour market adjustment relative to the cyclical decline in real GDP (Panel C). In the Great Recession, from peak to trough working time per employee decreased cyclically by 1.7%, a larger decline than during the Coronavirus Recession. But in the latest contraction the speed of the working-time reduction was faster than in the Great Recession. However, the major difference in the cyclical labour-market responses between the two recessions lies in the development of cyclical labour productivity in the United States. Cyclical labour productivity behaved slightly pro- to acyclical in the Great Recession and anticyclical in the Coronavirus Recession.

Overall, this section has shown that the German and US labour market reacted quite differently during the last two recessions. In Germany, internal flexibility dominated labour market adjustment, while in the United States it was external flexibility. This finding fits with the descriptions of the policy responses outlined above.

### Germany: internal flexibility

**Fig. 4:**
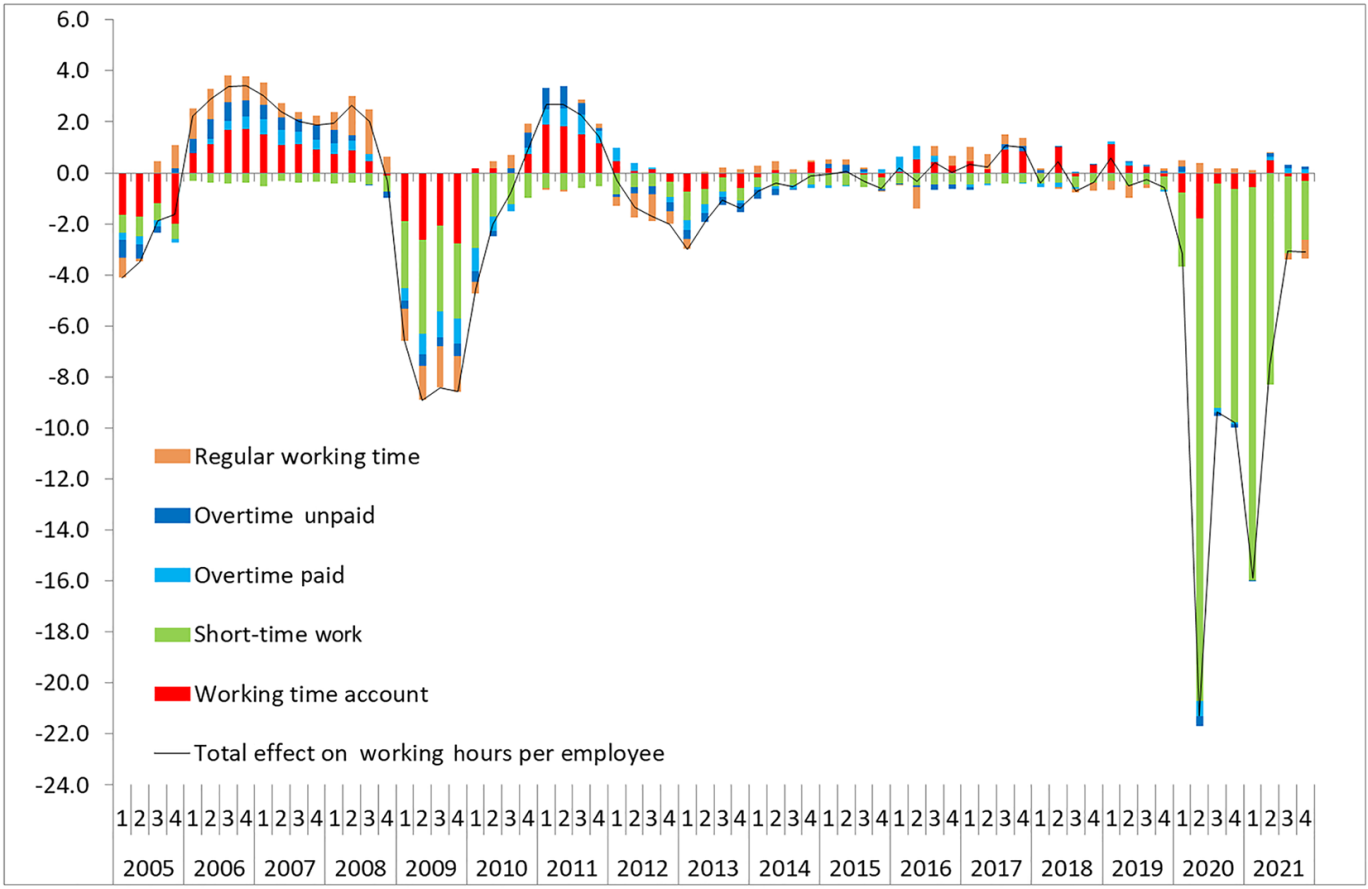
Components of cyclical changes in working hours per employee per quarter (2005–2021). The term ‘cyclical’ refers to the difference of actual and trend changes for each working-time instrument (if the series shows a trend). STW and WTA show no trend. The trend is constructed applying the Hodrick-Prescott filter with $\lambda =1 600$. All components are measured in working hours per employee per quarter. Sources: Institute for Employment Research (IAB) working time calculations; own calculations

In Germany, several instruments of internal flexibility are available at the establishment level to temporarily adjust the number of hours worked per employee in response to changes in the economic environment, such as overtime, working-time accounts, temporary changes in regular working time and STW. Figure 4 therefore shows the development of cyclical working time per employee and its components regular working time, paid and unpaid overtime, STW, as well as WTA, again detrended with the HP-filter ($\lambda$ = 1600) if the component has a trend. Over the period from the beginning of 2005 to the end of 2020, working time and all its components follow a clear cyclical pattern. However, while all these components contributed to the safeguarding of employment during the financial crisis (Herzog-Stein et al. 2018), this is no longer the case in the Coronavirus Recession.

#### Short-time work (STW)

**Fig. 5:**
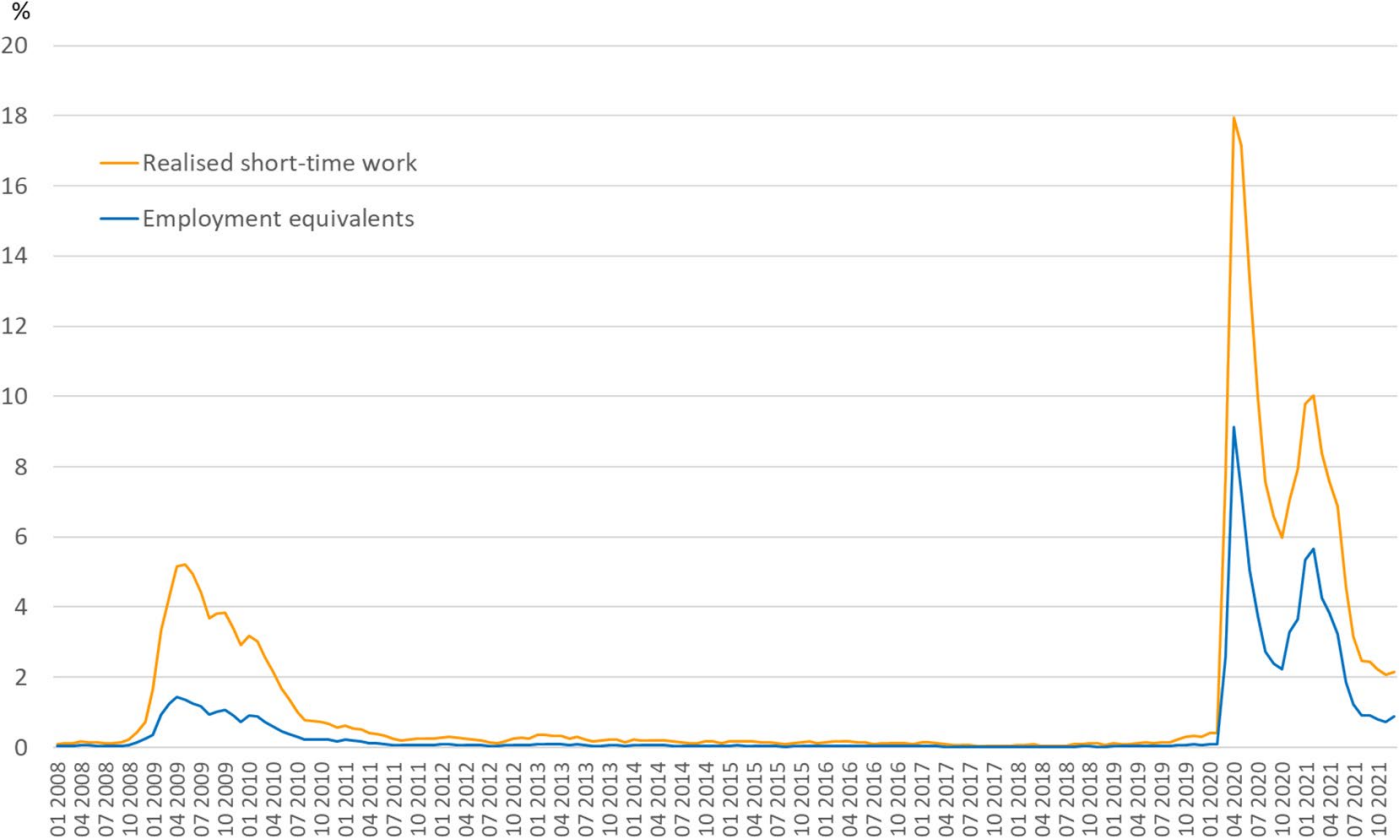
Short-time work and employment equivalents (2008–2020). Proportion of short-time workers (realised numbers or employment equivalents) in total employment subject to social security contributions. Sources: Federal Employment Agency; own presentation

In terms of safeguarding jobs, STW has two important dimensions: the number of workers in STW and the intensity of STW, i.e., the number of reduced working hours per short-time worker due to STW. Comparing the development of STW in both recessions, two aspects stand out particularly. First, policy makers reacted fast and made the use of STW more attractive for establishments immediately after the outbreak of the Covid-19 pandemic at the end of the first quarter 2020. This had the effect of introducing STW on a uniquely large scale in both dimensions of STW. In April 2020, the month with the highest incidence of STW in the Coronavirus Recession, almost 6 million, or 17.9% of all employees subject to social security contributions were in STW. The average loss of working time for a short-time worker was nearly 50%. In employment equivalents this corresponded to 9.1% of all employees subject to social security contributions (Fig. 5).

Although the number of employees in STW declined steadily after April 2020, there were still more employees in STW in October 2020 than at the peak of the Great Recession. As a result of the second wave of the Covid-19 pandemic, the number of workers in STW rose again from November 2020 and peaked in February 2021 before declining again.

Consequently, in the Coronavirus Recession there was a rapid cyclical reduction in average working time per worker of 2.4 h already in 2020q1 alone (relative to 2019q4). This is comparable in its magnitude to the cyclical working-time reduction induced by the use of STW from peak to trough in the whole Great Recession of 3.3 h per worker—of which 3.1 h were reduced in the first two quarters of 2009 relative to the last quarter in 2008.

Second, while the immediate response in STW was already comparable to the Great Recession, at the trough of the Coronavirus Recession in the second quarter 2020, STW reduced the average working time per worker by 18.4 h compared to the peak quarter 2019q4, more than five times the working-time reduction due to the use of STW in the Great Recession. On average, STW is accounting for around 89% of the total reduction in hours worked per worker from 2019q4 to 2020q2.

**Fig. 6:**
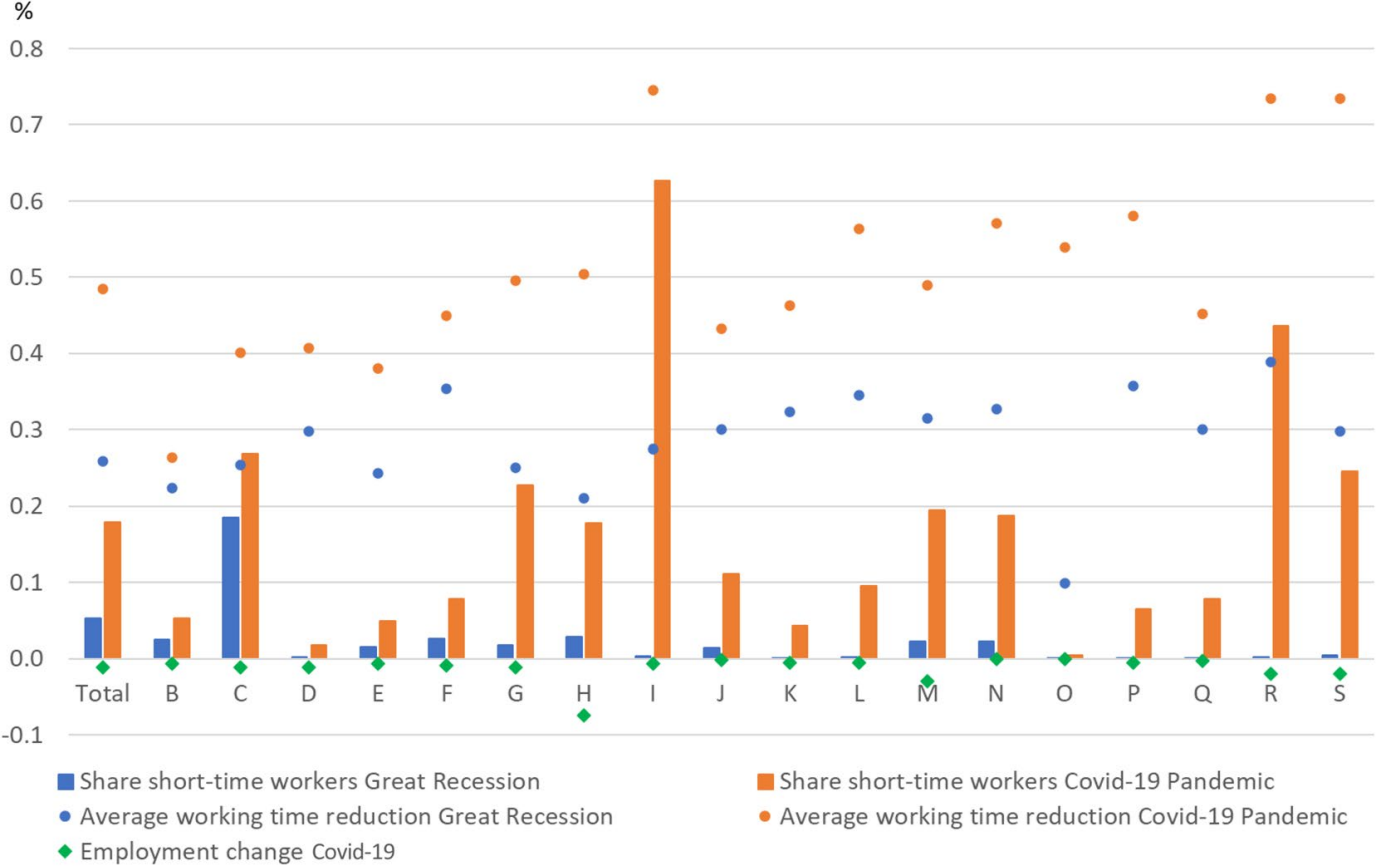
Share of recipients of short-time allowance, average working time reduction, and employment change by economic sector. B: Mining and quarrying; C: Manufacturing; D: Electricity, gas, steam and air conditioning supply; E: Water supply; sewerage, waste management and remediation activities; F: Construction; G: Wholesale and retail trade; repair of motor vehicles and motorcycles; H: Transportation and storage; I: Accommodation and food service activities; J: Information and communication; K: Financial and insurance activities; L: Real estate activities; M: Professional, scientific and technical activities; N: Administrative and support service activities; O: Public administration and defence; compulsory social security; P: Education; Q: Human health and social work activities; R: Arts, entertainment and recreation; S: Other service activities. Short-time work refers to the share of recipients of short-time allowance by economic sector in May 2009 and April 2020 respectively (columns). The intensity of STW refers to the average reduction in working time of a short-time worker (in %) due to STW (dots) and is calculated by dividing the employment equivalent by short-time workers. Change in employment (diamonds) refers to the sum of employment subject to social security contributions (seasonally adjusted) and exclusively marginally paid employees by economic sector from March to April 2020. Data on marginally paid employees by sectors are only available since 2020. Hence no seasonally adjusted data are available. Given that employment is not provided in each economic sector, employment changes of the sectors B, D, E, L, M, O, U, R, S, T are approximated by the corresponding average employment changes by the sums of B + D + E, L + M, O + U, R + S + T. Sources: Federal Employment Agency; own calculations

In the two recessions, employees subject to social security contributions were affected differently by STW in the individual economic sections (Fig. 6). A comparison between May 2009 and April 2020, the months with the highest incidence of short-time work in both downturns, shows that this time not only was the number of short-time workers significantly higher, but in the economy as a whole STW was used more heavily (columns in Fig. 6). While more than 80% of short-time workers were employed in manufacturing during the Great Recession, it was only about 31% during the Coronavirus Recession.^6^

In the Coronavirus Recession, STW is also used more intensively across all economic sections (dots in Fig. 6). In the total economy, the intensity of the use of STW in April 2020 was nearly twice as high as in May 2009. The average intensity of STW use was particularly high in the services sector, exceeding 70% in sections ‘Accommodation and food service activities’ (I), ‘Arts, entertainment and recreation’ (R), and ‘Other service activities’ (S). In the past, STW intensity of 100% was not common. In the Coronavirus Recession it was used only modestly, despite the severity of the crisis. According to Kruppe and Osiander (2020) using information on the individual STW intensity from a survey in May 2020, 24.1% of all STW-workers reported a loss in hours of 100%, but still more than half a loss in hours of only up to 50%.

In contrast to the importance of internal flexibility and especially the use of STW, external flexibility—unlike in the United States (see Sect. 3.2.1)—hardly played a role in Germany between March and April 2020. The overall change in employment, measured by the sum of employees subject to social security contributions and workers only marginal employed, was only about − 1% on average (diamonds in Fig. 6). Only in section H (Transportation & Storage) there is a substantial drop in employment of − 7.5%.

#### Working-time accounts (WTA)

Together with STW, they were the most important instrument of internal flexibility during the Great Recession. Like STW, the use of WTA at that time reduced the average working time per employee by 3.3 h in total or 0.7 h per quarter from peak to trough. In the Coronavirus Recession, the contribution of WTA to the temporary reduction in average hours worked per worker is this time much smaller than in the Great Recession. From peak to trough, WTA contributed 1.7 h, or on average 0.8 h per quarter, to the reduction in average hours worked per worker in the latest downturn.

At first glance, this is unexpected, as WTA became more common over time and 56% of all employees had WTA in 2016 (Ellguth et al. 2018). However, one possible explanation could be the respective economic dynamics in the boom periods before the two recessions.

In the upswing before the Great Recession, WTA were filled, providing firms with a considerable working-time-account buffer for the following downturn. In contrast, in the long boom period before the Coronavirus Recession, working time was closer to its long run trend with smaller cyclical variations. As a result, opportunities to increase the balances in the WTA were more limited than in the boom period before the Great Recession. Therefore, the working-time reductions due to WTA account only for 8% of total working-time reduction in the latest recession from 2019q4 to 2020q2.

#### Overtime

In general, paid and unpaid cyclical overtime vary between ± 1 h per quarter over the business cycle. Unpaid overtime was most important at the beginning of the considered period (Fig. 4). After the minor economic slowdown in Germany related to the so-called Euro Crisis from 2011q3 to 2013q1, it lost its relevance for cyclical fluctuations. Interestingly, unlike unpaid overtime, the cyclical variation of paid overtime continues after the Great Recession and can also be observed in the Covid-19 pandemic.

In the Coronavirus Recession, the contributions of paid and unpaid overtime to the cyclical reduction in working time from 2019q4 to 2020q2 on a quarterly basis (− 0.3 h vs − 0.2 h per quarter) is similar to that in the Great Recession (− 0.2 h and − 0.2 h per quarter), but together accounting only for less than 5% of the total working-time reduction per worker during that time period, in contrast to nearly 20% in the Great Recession.

#### Regular working time

Unlike in the Great Recession, there is not really a cyclical response in regular working time to reduce working hours in the Coronavirus Recession. The cyclical component of regular working time per worker even slightly increased average working hours per worker by on average 0.3 h from 2019q4 to 2020q2. Overall, this observation might be explained by the dominance of STW, which made further adjustments to working time unnecessary.

#### Summary

**Fig. 7:**
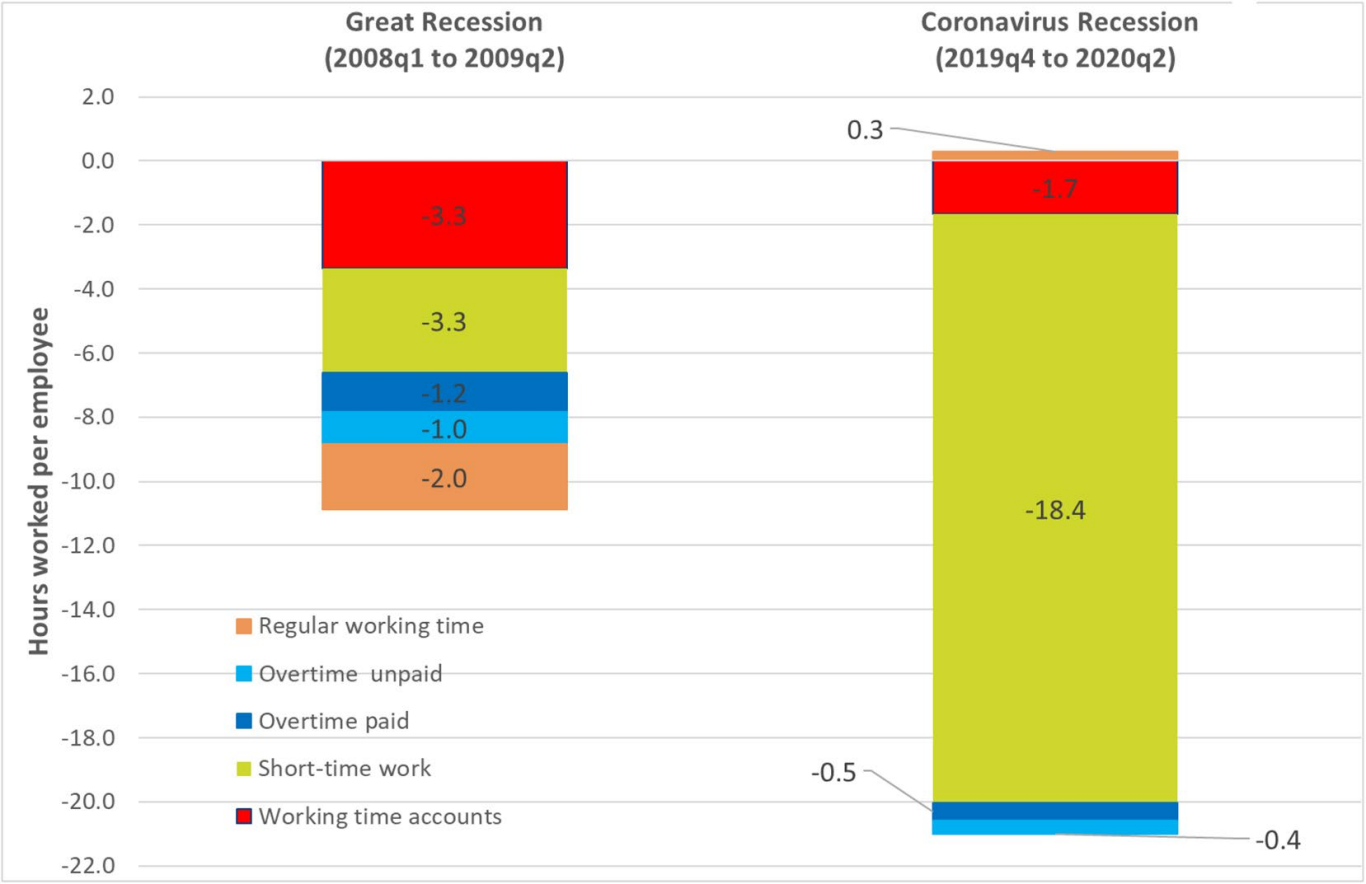
Contributions to the cyclical working-time reductions in the Great Recession and the Coronavirus Recession. The term ‘cyclical’ refers to the difference of actual and trend changes for each working-time instrument (if the series shows a trend). STW and WTA show no trend. The trend is constructed applying the Hodrick-Prescott filter with $\lambda =1 600$. All components are measured in working hours per employee. Sources: Institute for Employment Research (IAB) working time calculations; own calculations

In conclusion, although external flexibility again was of minor importance and instruments of internal flexibility played a crucial role in the safeguarding of employment in both the Great Recession and the Coronavirus Recession in Germany, a closer look at various working-time components shows marked differences between the two recessions. While in the Great Recession several instruments contributed markedly to the temporary decline in hours worked per worker, in the Coronavirus Recession STW is the instrument that has contributed by far the most to the reduction in working hours (Fig. 7).

In the Great Recession, STW and WTA contributed equally to the cyclical reduction in working time from peak to trough (− 3.3 h each). Paid and unpaid overtime and a temporary reduction in regular working hours both reduced cyclical working time by an additional two hours. In contrast, while most instruments responded as expected in the latest downturn, in absolute and in relative terms STW was by far the main driver to safeguard employment in the Coronavirus Recession (− 18.4 h). WTA was again the second most important instrument of internal flexibility used. However, its quantitative importance was smaller, reducing average working hours per employee by 1.7 h. The same is true for paid and unpaid overtime which together reduced average working hours by another 0.9 h. Reductions in regular working time do not contribute to the cyclical reduction in working time in the Coronavirus Recession. The observed dominance of STW in the attempt to safeguard employment in the Coronavirus Recession is in line with the made discretionary policy changes governing the use of STW. It is conceivable that the extended and simplified use of short-time work “crowded out” to some extent the use of other measures like e.g., WTA since already in March 2020 no negative balances on WTA were required anymore as eligibility criteria for the use of STW.

**Fig. 8:**
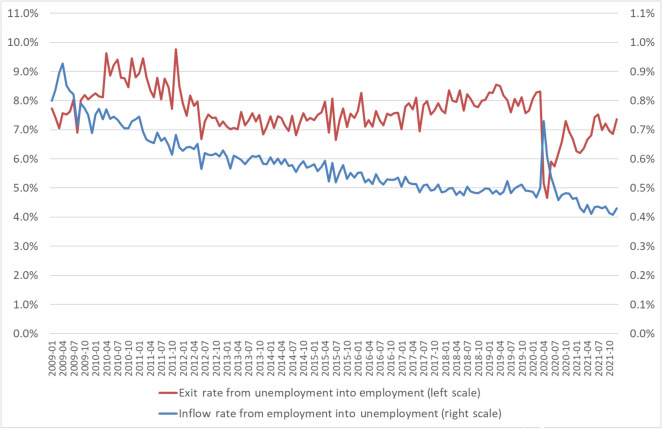
Monthly unemployment flows (2009 to 2021). The monthly inflow rate from employment into unemployment (national definition) is defined as the number of inflows from employment to unemployment in month t relative to the employment level in month t-1. The monthly exit rate from unemployment into employment is defined as the number of outflows from unemployment (national definition) to employment in month t relative to the unemployment level in month t-1. The numbers are seasonally adjusted. Sources: Federal Employment Agency; own calculations

Finally, the important impact of the use of STW on unemployment respectively employment is best seen by looking at the seasonally adjusted inflow rate from employment into unemployment and the exit rate from unemployment into employment on a monthly basis (Fig. 8). From February to April 2020 the inflow rate from employment into unemployment increased from 0.5 to 0.7% and declined than quickly back to 0.5% in June, while the exit rate from unemployment into employment decreased from 8.3 to 4.7% from February to May 2020 and did not reach its pre-pandemic level until the end of 2021. This can be seen as some indication that the massive use of STW was able to prevent large and prolonged flows from existing employment into unemployment.

### United States: external flexibility

Although STW programs exist in about half of the U.S. states and STW utilisation was much higher than in the past, the use of STW has overall not played a major role in the United States (Krolikowski and Weixel 2020). Here, the focus was rather on external flexibility. However, for the first time, the use of temporary lay-offs, i.e. laid-off individuals who expect to be recalled by their former employers (Gallant et al. 2020), was the prominent tool for dealing with the crisis.

**Fig. 9:**
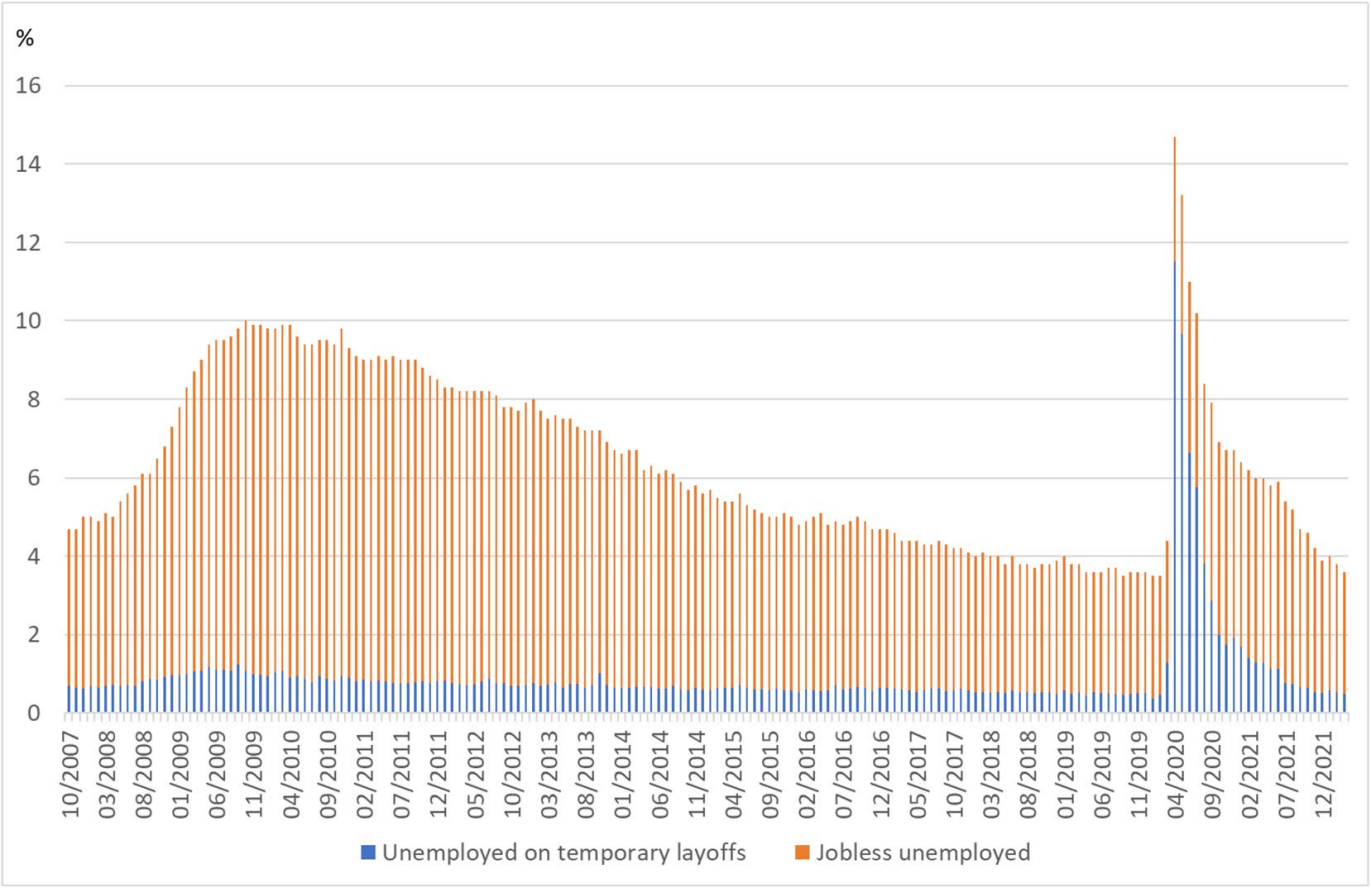
US unemployment rate: temporary layoffs and jobless unemployed. Jobless unemployed comprises job losers not on layoff, job leavers, reentrants to labour force and new entrants to labour force. Sources: U.S. Bureau of Labor Statistics (BLS), own calculations

While temporary unemployment has been between 0.4 and 1.2% throughout the years and even during the economic and financial crisis it played no prominent role with respect to the overall increase in unemployment, the share of workers on temporary layoffs jumped to 11.5% in April 2020, accounting for almost 80% of all unemployed persons (Fig. 9). While unemployment declines slowly during an economic recovery, the work-finding rate^7^ for the temporarily laid-off unemployed is usually twice as high as for the unemployed. Accordingly, unemployment fell faster this time than in previous recoveries (Hall and Kudlyak 2022). Thus, the rate of temporary unemployment halved from April to July 2020, while the jobless unemployment rate increased by 1.2 percentage points.

External flexibility via temporary lay-offs was the main means of the labour-market adjustment in the Coronavirus Recession, but complementary to this, companies also used some measures of internal flexibility by reducing the working hours of their employees. While unlike in Germany there are no detailed information on the average number of working hours lost per worker due to the Covid-19 pandemic in the United States, an additional survey conducted by the BLS beginning in May 2020 as part of the Current Population Survey provides a good insight into the extent to which workers in non-agricultural industry were affected by the crisis. In this survey, workers were asked whether they had been unable to work due to the pandemic in the last four weeks and whether they had received any kind of payment from their employers. Unfortunately, no information was asked about the form of compensation paid or the exact number of hours lost as a consequence of the pandemic. Thus, it is also not known whether workers who were compensated for working time lost due to the pandemic received any payments via one of the short-time work programs at the state level.

**Fig. 10:**
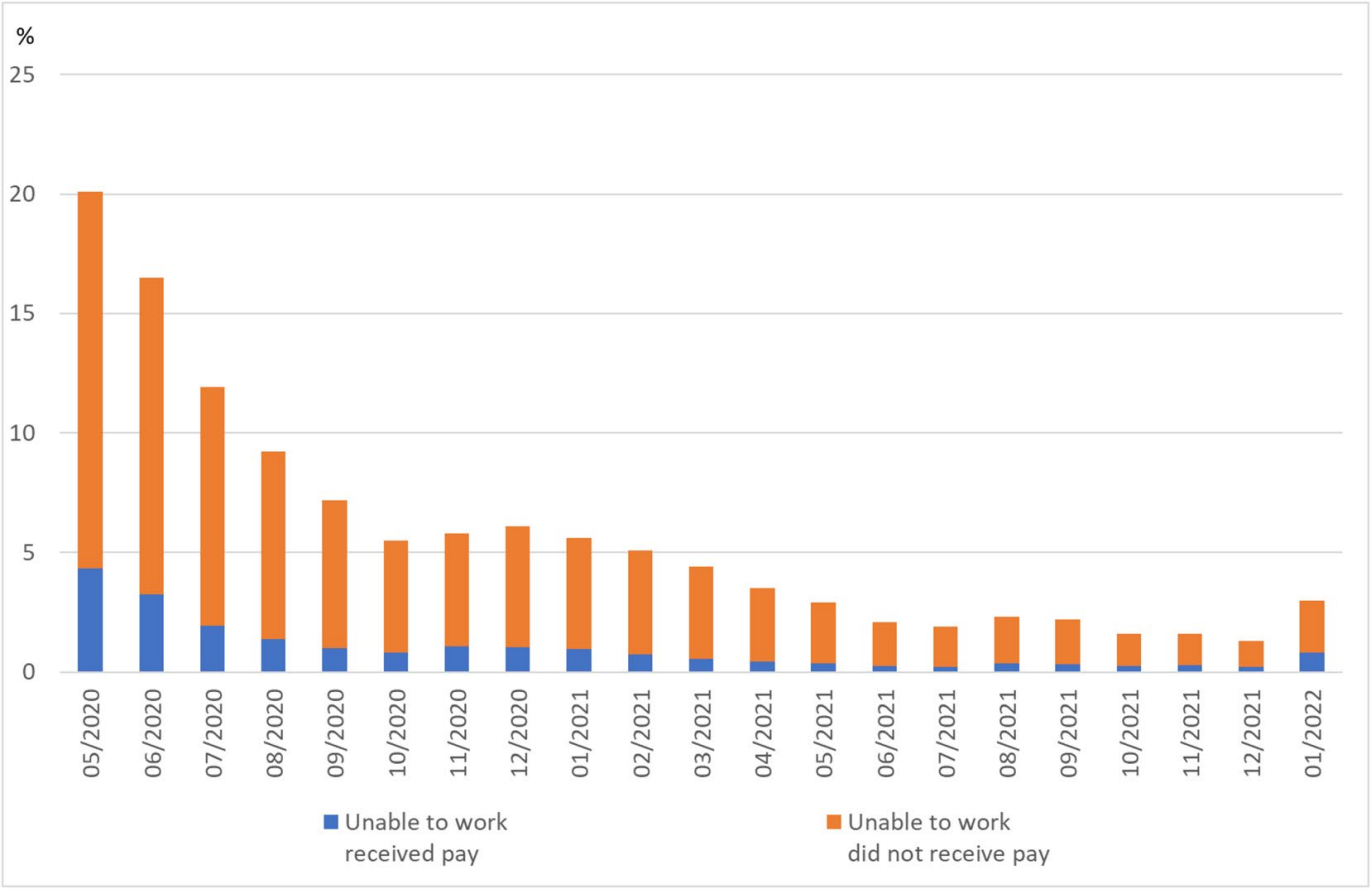
Proportion of persons in the United States unable to work due to lost business in the coronavirus pandemic. Supplemental data measuring the effects of the Coronavirus (COVID-19) Pandemic on the labour market. Persons unable to work at some point in the last 4 weeks because their employer closed or lost business due to the Coronavirus pandemic by receipt of pay from their employer for hours not worked and employment status. Sources: U.S. Bureau of Labor Statistics (BLS) Release “Effects of the Coronavirus (COVID-19) Pandemic on the Labor Market”, https://www.bls.gov/cps/effects-of-the-coronavirus-covid-19-pandemic.htm; own calculations

In May 2020, 20% of workers reported that they were affected by some kind of loss of working time,^8^ and in June and July 2020 still more than 15 respectively 10% of workers experienced some loss of working time (Fig. 10). The majority of them reported that they were not compensated by their employers. Only less than a quarter received some compensation. Therefore, the reduction respectively loss in working hours in the United States took place in a way that is quite different from the short-time allowance in Germany.

Overall, these information on unpaid as well as on compensated temporary working time losses fit together with the macroeconomic evidence for the United States presented in Sect. 3.1. As shown above there was some cyclical reduction in the average working time per employee from peak to trough of 1.0% in the Coronavirus Recession. If we take into account that the Coronavirus Recession was much shorter than the Great Recession, the average individual working-time reduction per quarter was stronger this time. Given that “job-losses have disproportionally hit the low-wage workforce” (Bateman and Ross 2021) it is also likely that the reported working-time losses were concentrated among the low-skilled. Since low-skilled workers generally have a lower hourly labour productivity, this would explain the anticyclical increase in labour productivity observed in the Coronavirus Recession (see Fig. 4D).

**Fig. 11:**
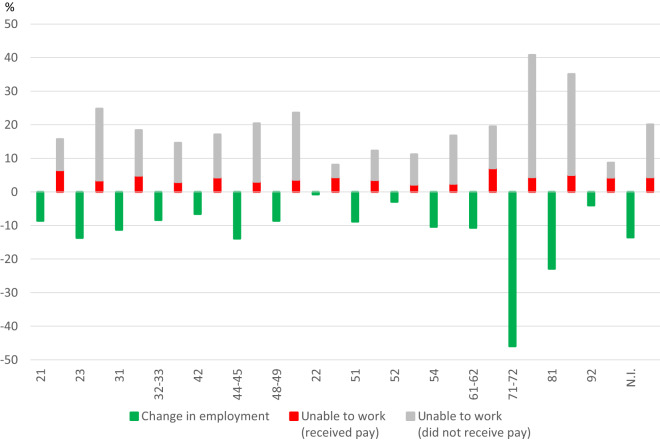
Proportion of persons in the United States unable to work (with and without compensation) due to lost business and change in employment by industry in April 2020. NAICS classification. 21 = Mining, quarrying, and oil and gas extraction, 23 = Construction, 31 = Durable goods manufacturing, 32–33 = Nondurable goods manufacturing, 42 = Wholesale trade, 44–45 = Retail trade,48–49 = Transportation and warehousing, 22 = Utilities, 51 = Information, 52 = Financial Activities, 54 = Professional & Business Services, 61–62 = Education & Health Services, 71–72 = Leisure & Hospitality, 81 = Other Services, 92 = Public administration, N.I. = Nonagricultural industries. Proportion of persons unable to work are from May 2020 which refers to the previous four weeks. Data for the change in employment refers to the monthly change from March to April 2020. Sources: U.S. Bureau of Labor Statistics (BLS) Release “Effects of the Coronavirus (COVID-19) Pandemic on the Labor Market”, https://www.bls.gov/cps/effects-of-the-coronavirus-covid-19-pandemic.htm, Current Employment Statistics (CES), own calculations

More details about the American way of dealing with the Coronavirus Crisis are revealed by looking closer at the change in employment and the share of workers affected by working hours lost due to the inability to work in different economic sections of the US economy (Fig. 11). Given that for economic sectors no data for temporary layoffs are available the change in employment is used instead to indicate the intensity with which employers were hit by job losses across economic sectors. As in Germany, the economic sections have been affected differently by the Covid-19 pandemic, the service sectors more than the industry.

Interestingly, a combination of layoffs and reductions in working hours dominates in all sectors of the economy. Hence, the dominance of external flexibility in the labour market adjustment in the United States does not imply that firms do not use measures of internal flexibility, too. The information from the economic sectors indicates that economic sectors that were hit hard by the Covid-19 pandemic relied on external as well as on internal flexibility in response to the Coronavirus Recession. There is a strong positive correlation between layoffs and working-time reductions with a correlation coefficient of 0.9: in economic sectors with a larger reduction in employment there is also a larger share of workers with a loss of working hours due to the inability to work. However, this positive relationship is driven by the positive correlation between employment reductions and working-time losses without compensation; there is no correlation between the magnitude of employment losses and the size of the share of workers with renumerated working-time losses. This suggests that, in contrast to the experience in Germany, in the United States the burden of labour market flexibility in the Coronavirus Recession is borne primarily by workers.

**Fig. 12:**
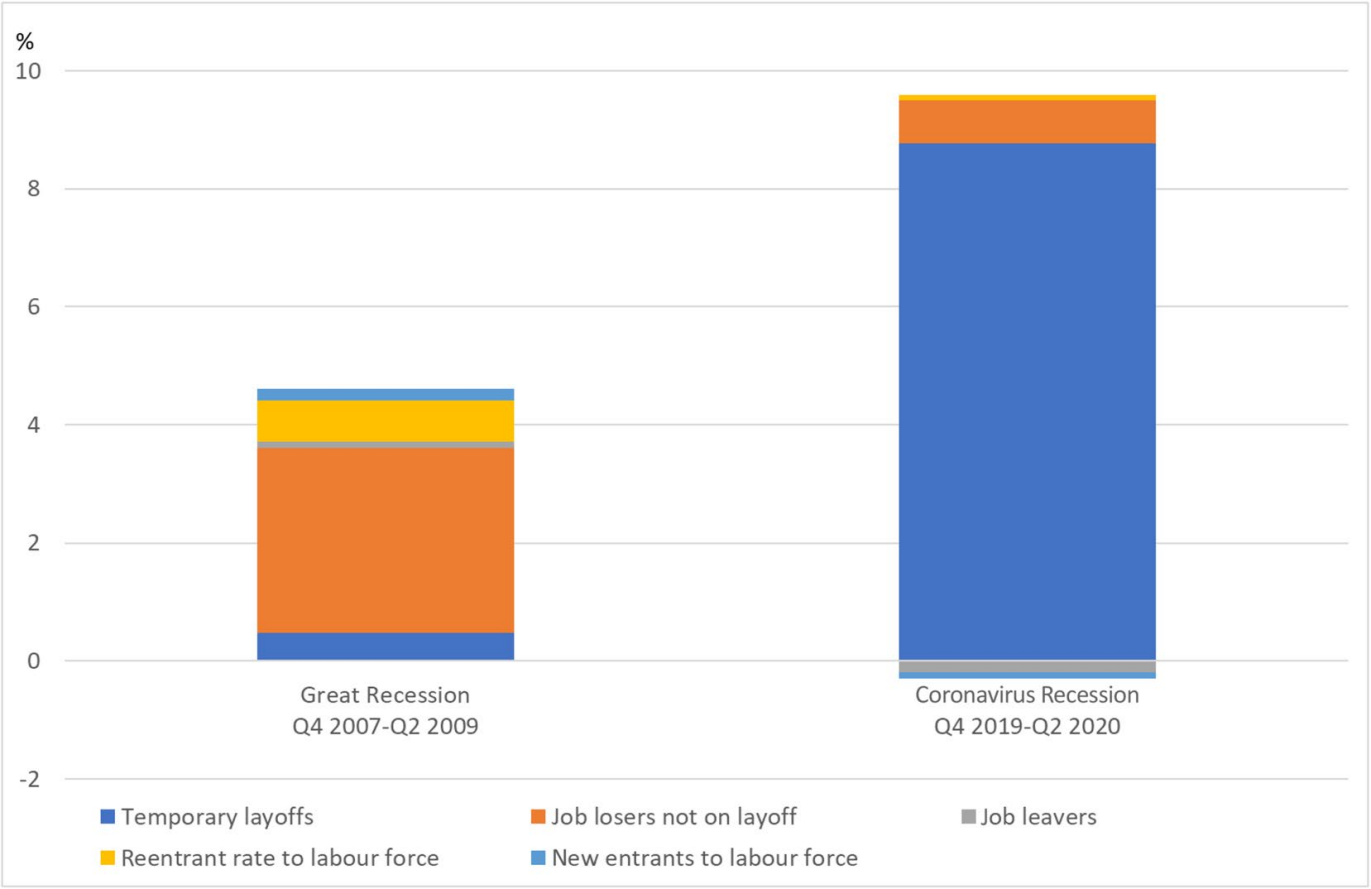
Contributions (in percentage points) of the components of US unemployment in the Great Recession and the Coronavirus Recession. Jobless unemployed comprises job losers not on layoff, job leavers, reentrants to labour force and new entrants to labour force. Sources: U.S. Bureau of Labor Statistics (BLS); own calculations

In conclusion, the United States have relied again heavily on the use of external flexibility. However, there are also major differences in its response compared to the Great Recession as a breakdown of the change in the unemployment rate in the two crises reveals (Fig. 12).

Most of the change in the unemployment rate is determined by the number of employees losing their jobs, usually without being on recall. During the Great Recession, the unemployment rate rose by 4.5 percentage points. Only a small part of 0.5 percentage points was due to temporary layoffs. Another 3.1 percentage points of the increase in the unemployment rate was due to workers losing their jobs. The proportion of job leavers among the unemployed is hardly influenced by the business cycle and lies typically in a range between 0.5 and 0.6%. Hence, its contribution to the change in unemployment over time is negligible. Almost one percentage point of the increase was due to re-entrants and new entrants into the labour market during the Great Recession.

As shown in the analysis above the labour market response during the Coronavirus Recession was extraordinary and very different to the one observed in the Great Recession. For the first time, temporary layoffs played a prominent and dominant role in the United States. The unemployment rate rose by a total of 9.4 percentage points from peak to through, of which 8.8 percentage points were due to temporary layoffs and only 0.7 percentage points to workers who lost their jobs. Interestingly, fewer employees seemed to leave their job of their own accord, and no change in labour force entry was observable.

## Blind spots of the chosen strategy

Germany and the United States pursued different goals with their Coronavirus Crisis responses. Germany focused on employment protection via mechanisms designed to promote internal flexibility. The United States focused on a mixture of external flexibility and income protection. As strategy objectives were different from one another, the blind spots are likely to differ between the countries. Therefore, we discuss the challenges each strategy poses in this last section.

### Germany’s employment protection

For the success of Germany’s chosen strategy, the main goal was to secure existing employment relationships to save firm specific human capital, prevent unemployment, and to reduce future training costs.

The burden of job losses in the Coronavirus Recession was unevenly distributed in Germany. Job loss rates of employment subject to social security contributions, which enjoys the protection of the STW scheme, were less severe than the losses of marginal employment (Minijobs).

**Fig. 13:**
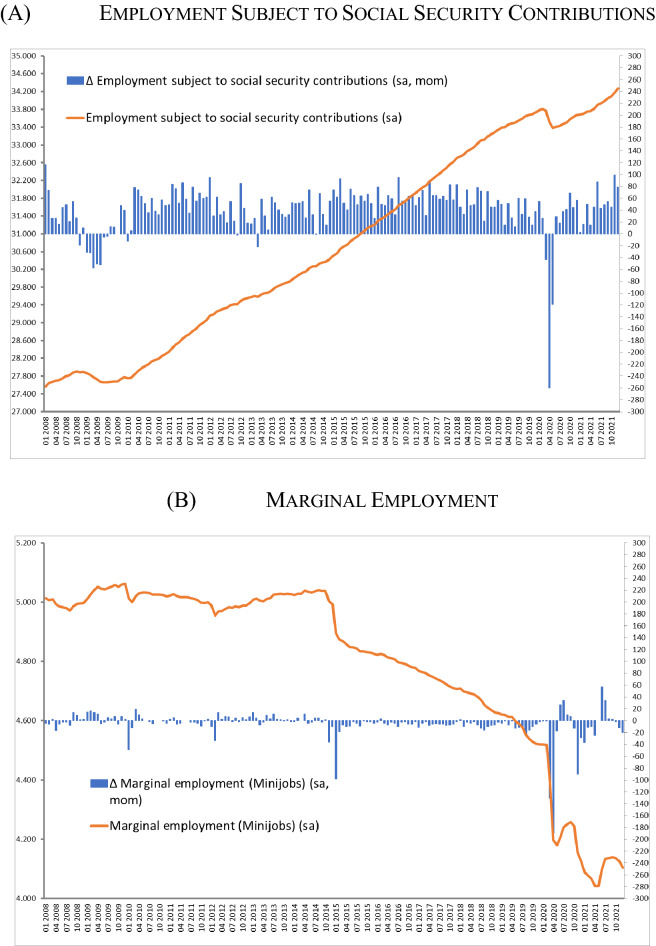
Different types of employment (2008–2021). Level (line, left scale) and change (columns, right scale) measured in 1000 persons (seasonally adjusted). Sources: Federal Employment Agency; Bundesbank; own presentation

Employment subject to social security contributions, the backbone of the German welfare state, decreased by around 450,000 jobs or 1.3% between March and May 2020 (see Fig. 13A). In line with their growth trends during the long boom before the outbreak of the Coronavirus Crisis, the recovery of employment subject to social security contributions was more dynamic than that of total employment in the summer and fall 2020.

Workers in marginal employment (Minijobs), who overwhelmingly work in the services sector were severely hit by the economic crisis due to the Covid-19 pandemic. In the months March to May 2020 the percentage decrease in marginal employment (− 7.5%) was five times as large as in employment subject to social security contributions (see Fig. 13B).

Even after accounting for the different growth trends of these two employment forms, marginal employment was more severely hit by the Coronavirus Crisis.^9^ Furthermore, while both employment subject to social security contributions and marginal employment started to recover in the summer months, marginal employment declined again with the second wave of the pandemic.

There are two obvious reasons for these remarkable employment patterns during the current Coronavirus Recession, which are interlinked. First, this time the services sector was much more hit by the economic crisis than during the Great Recession. Furthermore, in the Coronavirus Crisis the necessity to temporarily lock down and interrupt parts of economic activity to prevent the spread of infection cannot be overcome by stimulating aggregate demand. Rather, economic policies must try to sustain businesses, and hence employment, during these periods of (partial) lockdown and interruption of production processes.

**Fig. 14:**
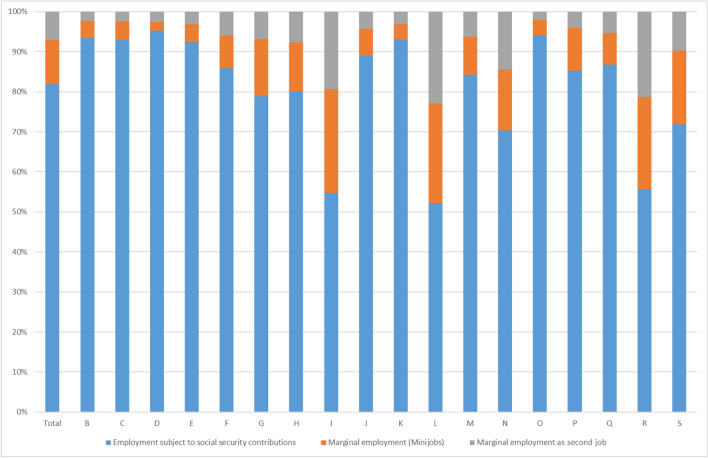
Composition of employment according to economic sectors (March 2020). B: Mining and quarrying; C: Manufacturing; D: Electricity, gas, steam and air conditioning supply; E: Water supply; sewerage, waste management and remediation activities; F: Construction; G: Wholesale and retail trade; repair of motor vehicles and motorcycles; H: Transportation and storage; I: Accommodation and food service activities; J: Information and communication; K: Financial and insurance activities; L: Real estate activities; M: Professional, scientific and technical activities; N: Administrative and support service activities; O: Public administration and defence; compulsory social security; P: Education; Q: Human health and social work activities; R: Arts, entertainment and recreation; S: Other service activities. Sources: Federal Employment Agency, own calculations

Second, STW, the major pillar of the government’s strategy to safeguard employment is not applicable to marginal employment. This left more jobs unprotected in the services sector, where marginal employment constitutes a larger share of total employment. In services sectors like accommodation and food service activities (section I), or arts, entertainment and recreation (section R), more than 40% of all employees were either working in marginal employment as their only or as their second job (Fig. 14).

Overall, since the services sectors were more severely affected by the Coronavirus Recession than by the Great Recession, some weaknesses in the approach to safeguard employment became visible. In contrast to employment subject to social security contributions, marginal employment as well as self-employment are not protected by the STW scheme.

### Differences in income protection in Germany

One advantage of STW besides securing existing employment relationships and firm specific human capital is that the income decrease during STW is less severe than with an immediate fall back to unemployment benefits. However, since income replacement rates of STW are comparable with the initial replacement rates of unemployment benefits, the impact of STW on income is dependent on the previous wage and the reduction in working time. This is also why Germany’s focus on employment protection via STW also helped with income protection. The widespread use of STW not only safeguarded employment, but also secured part of household income for households whose members were affected by STW.

One challenge of STW during the Coronavirus Recession was that the average short-time worker was very different from the average short-time worker in the Great Recession. The massive use of STW in other economic sections than manufacturing as well as the more intensive use of STW in general during the Coronavirus Recession have immediate income effects.

**Fig. 15:**
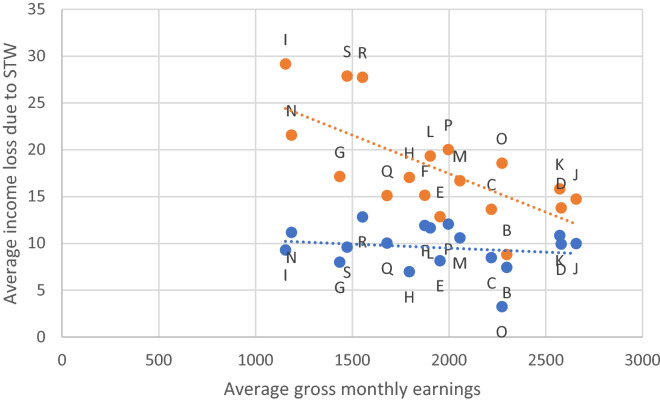
Earnings loss due to short-time work by economic sections. Blue (orange) dots indicate average income losses in the Great Recession (Coronavirus Recession) in %. For the comparison we use the information about the average amount of working time lost due to STW in the two crises which is provided by the Federal Employment Agency (Employment equivalent/number of short-time workers). Given that STW is paid on the basis of net earnings losses we calculate the impact of STW on average gross monthly earnings (SOEP) in Euro by using the Kurzarbeiterrechner on the assumption that the short-time worker is single, in tax class 1, without children. Sources: Federal Employment Agency, SOEP, Kurzarbeiterrechner (https://www.nettolohn.de/rechner/kurzarbeitergeld.html), own calculations

In Fig. 15 the average income losses due to STW in each economic section are plotted for the Great Recession and the Coronavirus Recession. Interestingly, whereas in the Great Recession there was no clear negative correlation between the level of earnings and the percentage earnings loss due to STW (blue dots), the situation during the Coronavirus Recession is completely different. We observe a clear negative correlation with a correlation coefficient of − 0.7 (orange dots). The lower the average earnings are in an economic section the higher is the percentage income loss due to STW. The difference is likely to be even more pronounced if we were to consider additional supplements of the short-time work allowance due to the employer which is more often paid in jobs with higher earnings (Pusch and Seifert 2020, Table 3). Pusch and Seifert (2020, Table 3) present the share of employees who receive a supplement to STW allowance. There is a positive correlation: The higher the average earnings in an economic sector, the higher the share of employees who receive a supplementary STW allowance from their employers. This implies that while the initial income loss was strongest in sectors with the lowest incomes, additional supplements to STW were concentrated among sectors with the highest incomes.

As pointed out before, not only short-time workers suffered income losses due to the loss of work. In general, all types of employment, who lost (temporary) part or all of their work, or even became unemployed, suffered income losses. Groups like the self-employed or workers in marginal employment were not entitled to STW or unemployment benefits. Walwei (2021) stresses that, in contrast to the previous crisis, employment associated with weak income security (marginal employment and solo self-employment) were particularly hard hit in the Coronavirus Crisis.

To conclude, STW did not protect employment and income of everyone equally. This is why further discretional measures were taken in a later stage of the Coronavirus Crisis to reduce the blind spots of the STW instrument. These additional discretionary policies as in example the one-off payments for children and an increase in STW replacement rates for long term recipients helped to cushion the income loss (Christl et al. 2021).

### Differences in income protection in the United States

There are two objectives for the success of the United States’ chosen strategy. First, the job loss should be temporary and not permanent. Second, the income of individuals should be protected despite the temporary job loss. As long as unemployment is only temporary and the focus is on re-employment, this approach has the potential to target individual’s particular in need of income stabilization.

Temporary layoffs were the dominant component of the unemployment increase (Fig. 9). However, they were rather unevenly distributed, and concentrated among high wage employees. While there was a rebound of employment for high wage earners, there were persistent job losses among low wage earners (Cajner et al. 2020; Chetty et al. 2020).

As discussed in Sect. 2, the CARES Act covered various instruments to secure income of individuals. Three policies to stabilize income directly or indirectly are of particular interest: The PPP, the Federal Pandemic Unemployment Compensation and Stimulus Checks.

While the initial idea of the PPP was to stabilise the income of individuals otherwise losing their jobs, stabilise companies’ financial flows, and support business owners through direct support for small business, the actual benefits had little to do with employment protection. Evaluations overall indicate that the program was untargeted and inefficient (Chetty et al. 2020; Autor et al. 2022). Even when it safeguarded employment, this was concentrated among employees in the top income quintile (Autor et al. 2022). However, as pointed out by Autor et al. (2022) this mainly results from the trade-off between a timely versus a targeted intervention under limited administrative capacities.

In contrast, the Stimulus Checks and the Federal Pandemic Unemployment Compensation proved to be an efficient tool to secure income. While large earning declines were more likely for low wage workers, both instruments together outweighed the otherwise resulting income losses (Larrimore et al. 2022) and stabilised income particularly at the lower end of the income distribution (Autor et al. 2022). Ganong et al. (2020) show that the majority of workers eligible for unemployment benefits between April and July 2020 had replacement rates above 100%. This lead to only a modest increase in poverty rates until the unemployment supplements expired (Parolin et al. 2020).

### Comparison of the strategies

The United States decided to insure workers’ incomes against the costs of job losses by increasing the generosity, eligibility criteria and eligibility period of unemployment benefits and other income support measures. In Germany, in contrast, labour hoarding was encouraged through STW programmes, which maintain employment relations between workers and firms. As a result, in the United States the unemployment rate (which is largely driven by temporary layoffs) and the non-employment rate rose sharply, as have the take up rates of STW in Germany.

Put differently, initial income support was stronger in the United States, but despite high levels of temporary layoffs, lead to a higher share of permanent job losses (Barrero et al. 2021), which in turn required more job search and reallocation activities. This was particularly true for lower wage earners. It remains to be seen whether these reallocation effects will be beneficial in the succeeding recovery. Germany, in contrast, secured existing employment relations at the expense of temporary income losses without protecting workers in marginal employment. While unemployment also leads to a persistent decline in wages, Giupponi et al. (2022) point out that Germany’s previous experience with STW indicates that wages of secured workers adjust to its pre-crisis level. The German approach therefore allowed to secure employment relations and human capital in the short run and secured income in the long run.

Overall, both countries protect individuals in weaker positions on the labour market (atypically employed and low wage earners) to a smaller extent through the chosen strategies. This is an indication of the segmentation of the labour market by wages and working conditions (Reich et al. 1973), which also reflect their lack of protection by labour market policies during the crisis.

Additionally, regardless of the labour market measure chosen, STW as well as the unemployment insurance carry the risk of moral hazard for labour market actors and are associated with social costs. Regarding STW, Cahuc et al. (2021) document moral hazard problems on the firm side by showing that firms with relatively low revenue shocks tend to reduce their employees’ working hours without actually safeguarding their jobs. In contrast, too generous unemployment benefits may induce moral hazard issues if they reduce search effort for new jobs (Schmieder et al. 2016). Despite the discussed blind spots and social costs, Giupponi et al. (2022, 50) conclude “…that short-time work can be an efficient and expedient way to attenuate the social costs created by “excess” layoffs in recessions”. Furthermore, since they argue for instance that both instruments cover different types of workers, STW and unemployment insurance can be valuable complements.

## Conclusion

The global Covid-19 pandemic hit the economies of Germany and the United States hard. Both countries experienced an economic downturn of similar magnitude. Interestingly, Germany and the United States pursued very different economic strategies to minimise the impact of the Coronavirus Crisis on the labour market. While Germany focused on safeguarding existing jobs through the use of internal flexibility measures, especially STW, the United States relied on a mix of external flexibility and income protection. This fact allowed us to examine more closely the German strategy of securing employment through internal flexibility and the German labour market development during the Coronavirus Recession by contrasting it with the chosen strategy and the labour market development in the United States.

Our analysis has shown that Germany responded to the economic shock with a massive temporary cyclical reduction in working hours, mainly through STW, on a historic scale and unemployment rose only moderately. In the United States, on the other hand, unemployment rose at an unprecedented rate, but unlike previous recessions, the nature of unemployment was quite different, being driven mostly by temporary layoffs. This allowed for a very fast recovery of unemployment in the United States—much faster than in previous recessions.

However, a closer look at the blind spots of the chosen strategies in Germany and the United States showed that despite the differences in the respective approaches, people in weaker labour market positions were less well protected by the chosen strategies. In Germany, marginally employed workers who lost their jobs were not protected by either STW or unemployment insurance. Moreover, low-income earners for whom the short-time allowance was not sufficient were additionally dependent on basic income support. In the United States, the Stimulus Checks and the Federal Pandemic Unemployment Compensation proved to be an effective instrument to secure income, especially for low wage earners. But they were even less protected from job losses and suffered disproportionately.

## Data Availability

The data that support the findings of this study are available from the *Hans-Böckler-Stiftung* and the *Research Data* Center *of the Socio-Economic Panel* but restrictions apply to the availability of these data, which were used under license for the current study, and so are not publicly available. Data are however available from the authors upon request and with permission of the *Hans-Böckler-Stiftung* and the *Research Data* Center *of the Socio-Economic Panel.*

## Footnotes

1 For more details on the difference between internal and external (numerical) flexibility see for example Herzog-Stein and Zapf (2014).

2 To enable a consistent comparison between the two countries, we use the unemployment rate as defined by the International Labour Office (ILO). For Germany, the monthly development of this unemployment rate during the Covid-19 pandemic differs with respect to the timing and the magnitude of the increase from the unemployment rate of the Federal Employment Agency, which is defined differently.

3 This view is also documented in Giupponi et al. (2022).

4 Throughout this business-cycle analysis whenever we use the logarithmic transformation of a time series *x*, growth rates are approximated by differences in log points, i.e., the growth rate of *x* in per cent is ${g}_{x}\approx \Delta \mathrm{ln}x\times 100.$ Growth of 1 log point is equal to a growth rate of approximately 1 per cent. The exact relationship is ${g}_{x}=100*\left(exp\left(\Delta \mathrm{ln}x\right)-1\right).$ For simplicity we always speak of percentage changes.

5 Both institutions determine the peaks and troughs of economic activity separately on a monthly and a quarterly basis. In general, the peak or trough quarter contain the peak or trough month. However, the determination of the peak of economic activity before the Coronavirus Recession is an exception. Both, NBER and GCEE determine February 2020 as the latest peak month but the fourth quarter 2019 as the peak quarter.

6 With respect to employment subject to social security contributions the employment structure of the German economy has changed only moderately since the Great Recession. The employment share in manufacturing (section C) has decreased from 23.3 to 20.8%. In turn, the employment share in the services sector (sections G-N) has risen.

7 The job-finding rate is often used to describe the labour market tightness. Hall and Kudlyak (2022) use the work-finding rate instead in the context of the Covid-19 pandemic given that unemployed on temporary layoffs are not obliged to look for a job while waiting to be recalled. Some of them might nevertheless take a new job rather than waiting to be recalled by the previous employer.

8 Given that the survey asks about the employment situation in the previous four weeks, the May survey probably covers most of the employment situation in April 2020, the month with the highest crisis impact.

9 Marginal employment exhibits a marked negative growth trend since the introduction of the general legal minimum wage at the beginning of 2015.
